# A Method for Subsampling Terrestrial Invertebrate Samples in the Laboratory: Estimating Abundance and Taxa Richness

**DOI:** 10.1673/031.010.2501

**Published:** 2010-03-25

**Authors:** Mahmut Doğramaci, Sandra J. DeBano, David E. Wooster, Chiho Kimoto

**Affiliations:** Department of Fisheries and Wildlife, Hermiston Agricultural Research and Extension Center, Oregon State University, Hermiston, OR 97838

**Keywords:** pitfall traps, laboratory sampling techniques

## Abstract

Significant progress has been made in developing subsampling techniques to process large samples of aquatic invertebrates. However, limited information is available regarding subsampling techniques for terrestrial invertebrate samples. Therefore a novel subsampling procedure was evaluated for processing samples of terrestrial invertebrates collected using two common field techniques: pitfall and pan traps. A three-phase sorting protocol was developed for estimating abundance and taxa richness of invertebrates. First, large invertebrates and plant material were removed from the sample using a sieve with a 4 mm mesh size. Second, the sample was poured into a specially designed, gridded sampling tray, and 16 cells, comprising 25% of the sampling tray, were randomly subsampled and processed. Third, the remainder of the sample was scanned for 4–7 min to record rare taxa missed in the second phase. To compare estimated abundance and taxa richness with the true values of these variables for the samples, the remainder of each sample was processed completely. The results were analyzed relative to three sample size categories: samples with less than 250 invertebrates (low abundance samples), samples with 250–500 invertebrates (moderate abundance samples), and samples with more than 500 invertebrates (high abundance samples). The number of invertebrates estimated after subsampling eight or more cells was highly precise for all sizes and types of samples. High accuracy for moderate and high abundance samples was achieved after even as few as six subsamples. However, estimates of the number of invertebrates for low abundance samples were less reliable. The subsampling technique also adequately estimated taxa richness; on average, subsampling detected 89% of taxa found in samples. Thus, the subsampling technique provided accurate data on both the abundance and taxa richness of terrestrial invertebrate samples. Importantly, subsampling greatly decreased the time required to process samples, cutting the time per sample by up to 80%. Based on these data, this subsampling technique is recommended to minimize the time and cost of processing moderate to large samples without compromising the integrity of the data and to maximize the information extracted from large terrestrial invertebrate samples. For samples with a relatively low number of invertebrates, complete counting is preferred.

## Introduction

A common problem facing entomologists and ecologists working with invertebrate communities is dealing with the sheer number of invertebrates usually associated with most invertebrate sampling techniques. Most field sampling techniques generate samples with hundreds to thousands of invertebrates (New 1998), and investigators are faced with the daunting task of processing samples in the laboratory. This process usually includes sorting invertebrates from debris, and then counting and identifying them to the desired taxonomic level. Thus, the laboratory processing of invertebrate samples associated with community ecology and biodiversity studies is costly and time consuming. One solution to this problem is to subsample, whereby investigators process and identify a random portion of the sample (Vinson and Hawkins 1996).

Most research on subsampling techniques for invertebrates has been conducted in the context of aquatic biomonitoring studies, which use macroinvertebrates to assess the health or biological integrity of aquatic ecosystems (e.g., Courtemanch 1996; Walsh 1997; Doberstein et al. 2000; Ostermiller and Hawkins 2004). Because of the extensive use of aquatic macroinvertebrates as bioindicators of stream quality, and the large numbers of invertebrates associated with these samples, the use of subsampling techniques in this field is widespread. For example, a survey conducted by Carter and Resh (2001) showed that 74% of the methods used by U.S. state agencies employed subsampling techniques in the laboratory, and the standard operating procedure within the US Environmental Protection Agency's Rapid Bioassessment Protocols includes laboratory subsampling (Barbour et al. 1999).

In contrast to research on subsampling techniques for aquatic invertebrates, few studies have examined subsampling techniques for terrestrial invertebrates (see Corbet 1966, for an exception). This is true, even though terrestrial field techniques, like aquatic ones, can collect large numbers of invertebrates (Corbet 1966; New 1998). Yet with the growth of fields such as conservation biology and applied ecology, the number of studies examining terrestrial invertebrate biodiversity has increased rapidly. Recent studies in ecosystems ranging from forests to grasslands have involved collecting thousands to tens of thousands of invertebrates, even with relatively little sampling effort in the field (e.g., DeBano 2006; Brosi et al. 2007; Hilt et al. 2007; Wenninger and Inouye 2008; Kennedy et al. 2009). Laboratory processing of such samples is costly and time-consuming, and the common practice of counting all terrestrial invertebrates collected in samples limits the number of ecological and biodiversity studies that can be undertaken, which, in turn, effectively limits knowledge in these areas. Therefore it is crucial to develop a standard subsampling strategy for terrestrial invertebrates. In this study, the effectiveness of a standardized subsampling technique that would be simple, efficient, and effective in describing basic attributes of terrestrial invertebrate communities was investigated. The specific objectives of this study were to: 1) develop an apparatus specially designed for subsampling invertebrate samples collected with two common terrestrial field techniques, pitfall traps and pan traps; 2) investigate the accuracy of a fixed area subsampling method in estimating the total abundance of all invertebrates in a sample; and 3) determine the method's accuracy in estimating total taxonomic richness at the order or family level.

## Materials and Methods

Terrestrial invertebrate samples from plastic pan traps (55 × 37 × 15 cm) were collected in the summers of 2006 and 2007 in riparian areas of northeastern Oregon, and samples from 550 ml pitfall traps were collected in the summer of 2007 from grassland sites in the Zumwalt Prairie in northeastern Oregon. Both types of traps were filled with soapy water and left open for one week The contents of traps were poured through a sieve with a 500 µm mesh size in the field and samples were stored in 75% alcohol until processed in the laboratory.

**Figure 1.**
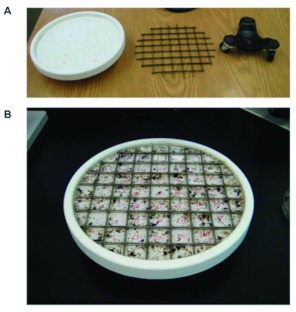
The subsampling apparatus: a) the turntable plate that holds the sample, the metal grid, and the three-wheel dolly; and b) an invertebrate sample prepared for subsampling. High quality figures are available online.

A subsampling apparatus was constructed using a plastic plate with a metal frame (Figure 1). The plastic plate formed the subsampling arena and consisted of a turntable or “Lazy Susan” plate (MadeSmart Housewares Inc., www.madesmart.com). A divided metal frame that fit inside the turntable was built from thin, scrap metal strips (6 × 1 mm). The outer diameter of the sampling arena was 25.4 cm and the inner diameter was 22.9 cm. The metal frame was built to fit inside the subsampling arena, and had 45 complete cells, with each cell measuring 2.54 × 2.54 cm (6.45 cm2). The total area inside of the plate was 412 cm2, or the equivalent to 63.6 subsampling cells. For simplicity, subsampling was limited to complete cells. The sampling plate was placed on a three-wheel dolly (Shepherd Hardware Products LLC, www.shepherdhardware.com) to facilitate easy movement of the plate under a stereomicroscope during the subsampling process.

To subsample, the following technique was used. Each sample was poured into a sieve with a 4 mm mesh size to remove large specimens, which were retained in the sieve. The portion of the sample that passed through the 4 mm mesh sieve was retained by a 0.02 mm mesh sieve. Large specimens were counted and identified, and the time taken for this process was recorded. The remaining sample was poured into the subsampling plate and dispersed with a small brush to provide an even distribution of invertebrates inside the subsampling plate. The subsampling frame was then placed inside the plate and invertebrates in 16 randomly selected cells (25% of the total area of the plate) were counted and identified, typically to order or family. The amount of time taken to count and identify invertebrates in each cell was also recorded. After 16 subsamples were taken, a quick scan was conducted of the remaining sample on the plate for individual taxa (to the level of order or family) that had not been found during sorting of the large invertebrates or in any of the 16 subsamples. The presence or absence of these taxa was recorded and used to calculate taxa richness. Each of the remaining individuals in the sample were then counted and identified to obtain the true number of individuals and taxa richness in the sample. The time necessary to complete processing of the entire sample was recorded. Three individuals, each with extensive experience in processing samples from the two studies, were involved in processing both types of samples. There were no obvious biases in the time taken or accuracy of identification among individuals.

To obtain an estimate of the total number of individuals in a sample, and the number in each taxon based on the subsampling effort, the average number of individuals per cell was calculated for 1–16 subsamples. That number was multiplied by 63.6 cells per plate. That estimate was divided by the actual number in the sample (minus the number of large specimens removed in the first phase) to obtain a “percent accuracy” score. Percents under or over 100%) indicate underestimates and overestimates, respectively.

To investigate whether the effectiveness of the subsampling method varied with the total number of individuals in the sample, samples were classified into three general categories based on overall abundance of individuals: low abundance samples had < 250 individuals, moderate abundance samples had 250–500 individuals, and high abundance samples had > 500 individuals. In each abundance category (low, moderate, and high), 10 samples were examined for each type of sampling method (pitfall and pan traps).

To compare the means of abundance and richness, 95% confidence intervals were used for estimates derived using from 1 to 16 subsamples and the means of those variables after processing the entire sample. Nonoverlapping confidence intervals indicated statistically significant differences. The mean time spent processing samples with 10 and 16 subsamples was compared with the mean time spent processing the entire sample using analysis of variance (ANOVA). Separate analyses were conducted for low, moderate, and high treatments for pitfall and pan traps. Means that were significantly different at α = 0.05 were compared using a least significant difference (LSD) test. Means in the text are reported ± one standard error.

## Results

### Pan traps

**Figure 2.**
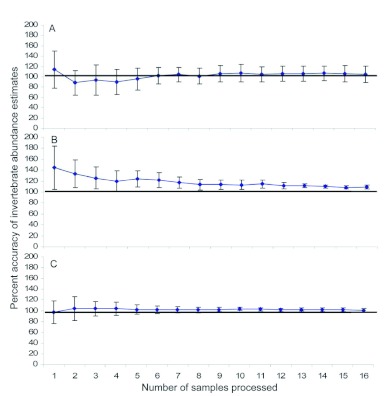
Percent accuracy of invertebrate abundance estimates produced by the second phase of the subsampling procedure for a) low, b) moderate, and c) high abundance invertebrate samples collected with pan traps. The solid horizontal line delineates the 100% accuracy level. Error bars denote 95% confidence intervals, and non-overlapping confidence intervals indicate statistically different means. High quality figures are available online.

A total of 27,663 invertebrates were counted in the 30 pan trap samples. The number of individuals found in low abundance samples ranged from 122 to 237 invertebrates, with a mean of 178 ± 12; moderate abundance samples ranged from 286 to 375 invertebrates, with a mean of 336 ± 10; and high abundance samples ranged from 676 to 5,337 invertebrates, with a mean of 2,193 ± 514 individuals. After taking 16 subsamples, the number of invertebrates in low and moderate abundance samples was overestimated by less than 10% (Figure 2a, b). The number of invertebrates in high abundance samples was estimated even more accurately; after 16 subsamples, accuracy was 101% (Figure 2c). There was no appreciable improvement in the accuracy or precision of abundance estimates for low, moderate, or high abundance pan trap samples associated with subsampling more than 10 cells (or 16% of the area in the plate) (Figure 2a, b, c).

**Figure 3.**
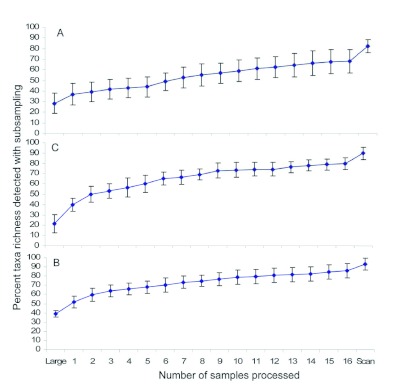
Percent taxa richness of invertebrates detected with the three phases for a) low, b) moderate, and c) high abundance invertebrate samples collected with pan traps. “Large” denotes the number of taxa detected in the first phase of the subsampling method, and “Scan” denotes the number of additional taxa detected during the third phase. Error bars denote 95% confidence intervals, and non-overlapping confidence intervals indicate statistically different means. High quality figures are available online.

The taxa found in pan traps are listed in Table 1. Taxa richness of pan trap samples corresponded to the size of the sample; mean taxa richness in low, moderate, and high abundance samples was 13.6 ± 0.9, 15.5 ± 0.9, and 18.7 ± 0.6, respectively. Initially, the percent taxa richness detected rapidly increased with increasing number of subsamples, but the rate of increase declined after taking approximately eight subsamples (Figure 3a, b, c); on average, less than two additional taxa were detected in samples after processing subsamples 9–16. The quick scanning procedure detected one or two more taxa than found after subsampling all 16 cells. On average, using the three-phase protocol and subsampling all 16 cells detected 82% of the taxa in low abundance samples, 90% of the taxa for moderate abundance samples, and 93% of the taxa for high abundance samples (Figure 3a, b, c).

Of the 30 taxa identified in pan traps, 14 taxa were common (found in more than 50% of all 30 samples, Table 1). Only two of these common taxa, Formicidae and adult Trichoptera, were missed in more than 15% of the samples. Only four relatively rare taxa were not detected by the subsampling technique in 50% or more of the samples in which they were present (Table 1).

**Table 1.**
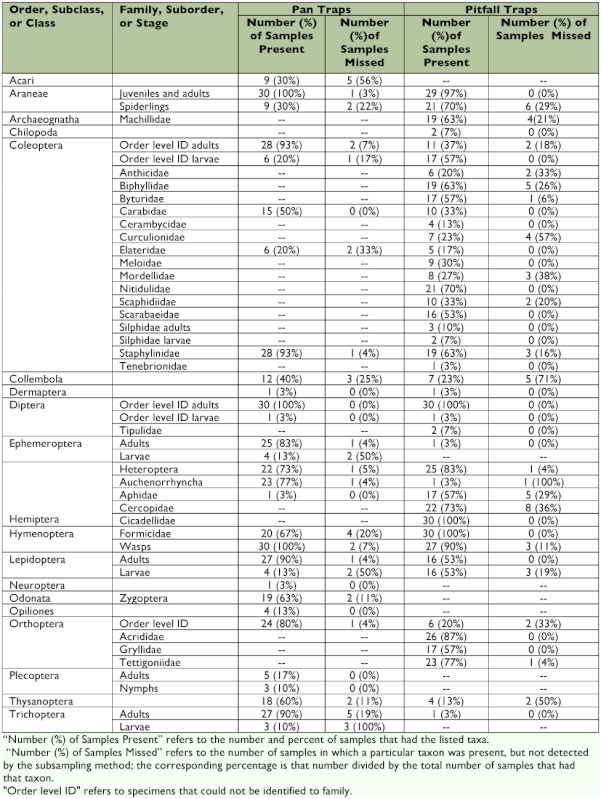
List of taxonomic groups identified in pan and pitfall traps.

### Pitfall traps

**Figure 4.**
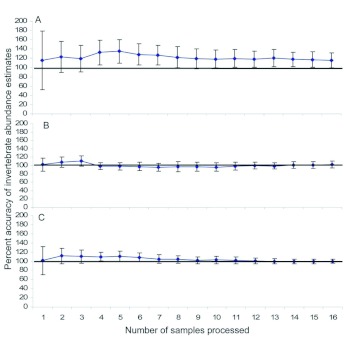
Percent accuracy of invertebrate abundance estimates produced by the second phase of the subsampling procedure for a) low, b) moderate, and c) high abundance invertebrate samples collected with pitfall traps; the solid horizontal line delineates the 100% accuracy level. Error bars denote 95% confidence intervals, and non-overlapping confidence intervals indicate statistically different means. High quality figures are available online.

A total of 12,195 invertebrates were counted in the 30 pitfall trap samples. Although low and moderate pitfall trap samples had similar numbers of invertebrates compared to pan traps, high abundance pitfall samples contained fewer invertebrates than high abundance pan trap samples. Number of invertebrates ranged from 93 to 164 for low abundance samples, with a mean of 131 ± 8; from 314 to 384 for moderate abundance samples, with a mean of 354 ± 8; and from 504 to 813 for high abundance samples, with a mean of 662 ± 33. The number of invertebrates in low abundance pitfall traps was overestimated by the subsampling procedure by almost 20% (Figure 4a).

However, estimates of the number of individuals in moderate and high abundance samples were highly accurate; the accuracy of estimation for both types of samples was approximately 100% after taking 10 subsamples (Figure 4b, c). There was no appreciable improvement in the accuracy or precision of abundance estimates for low, moderate, or high abundance samples associated with sampling more than 10 cells (Figure 4a, b, c).

**Figure 5.**
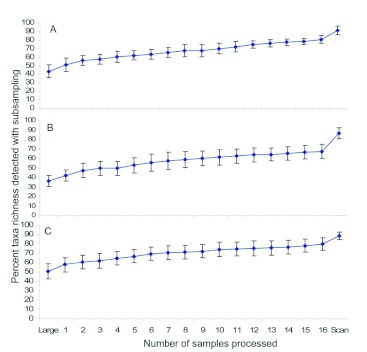
Percent taxa richness of invertebrates detected with the three phases for a) low, b) moderate, and c) high abundance invertebrate samples collected with pitfall traps. “Large” denotes the number of taxa detected in the first phase of the subsampling method, and “Scan” denotes the number of additional taxa detected during the third phase. Error bars denote 95% confidence intervals, and non-overlapping confidence intervals indicate statistically different means. High quality figures are available online.

Similar to pan trap results, taxa richness for pitfall samples increased with increasing numbers of subsamples, but the rate of increase declined after taking approximately eight subsamples (Figure 5a, b, c). On average, processing subsamples 9–16, quick scanning, and complete processing of the samples each added approximately two additional taxa to the total taxa richness. On average, using the three-phase protocol and subsampling all 16 cells detected 91% of the taxa in low abundance samples, 87% of the taxa for moderate abundance samples, and 89%) of the taxa for high abundance samples (Figure 5a, b, c). Taxa richness found after complete processing corresponded to the size of the sample; mean taxa richness in low, moderate, and high abundance samples of pitfall traps was 15.4 ± 0.9, 18.6 ± 1.2, and 23.7 ± 1.0, respectively.

Of the 43 taxa identified in pitfall traps, 21 taxa were common (i.e., found in more than 50% of all 30 samples, Table 1). Six of these taxa (Cercopidae, Aphidae, spiderlings, Biphylidae, Machillidae, and Lepidoptera larvae) were missed in more than 15% of the samples. Three taxa, a relatively rare Auchenorrhyncha taxon, and Collembola and Curculionidae, were not detected by the subsampling technique in more than 50% of samples.

### Time savings associated with subsampling

The first and third phases of the subsampling procedure are the least time consuming (Table 2).

The first phase (separating, sorting, and identifying large invertebrates from samples) took, on average, less than 10 min for pan trap samples and less than 12 min for pitfall trap samples, with larger samples taking more time for this phase (Table 2). The third phase (quick scanning) took, on average, 6–7 min for pan trap samples and 4 min for pitfall trap samples, and showed little to no variation with respect to sample size (Table 2).

The second phase (subsampling individual cells) was the most time-consuming step and was more variable with respect to sample type and size. For pan trap samples, the second phase for low and moderate abundance samples required approximately the same amount of time to be processed (16–38 min for sampling 10–16 cells; Table 2). However, the second phase for high abundance pan trap samples required approximately a four-fold increase in time (58–93 min for 10–16 cells) compared to low and moderate abundance samples. The entire three-phase subsampling procedure took 38–109 min for 16 cell counts and 28–74 min for 10 cell counts (Table 2). This is compared to 94–383 min for counting the entire sample. The time required to count the entire sample was significantly greater than the time required to process samples using 10 or 16 subsamples for all size categories (Table 2). Taking 16 subsamples saved, on average, approximately 1 hour per sample for low and moderate abundance pan trap samples and more than 4 hrs per sample for high abundance pan trap samples, compared to complete counting of the entire sample. An additional 10–35 min were saved per sample by taking 10 subsamples instead of 16 (Table 2).

**Table 2.**
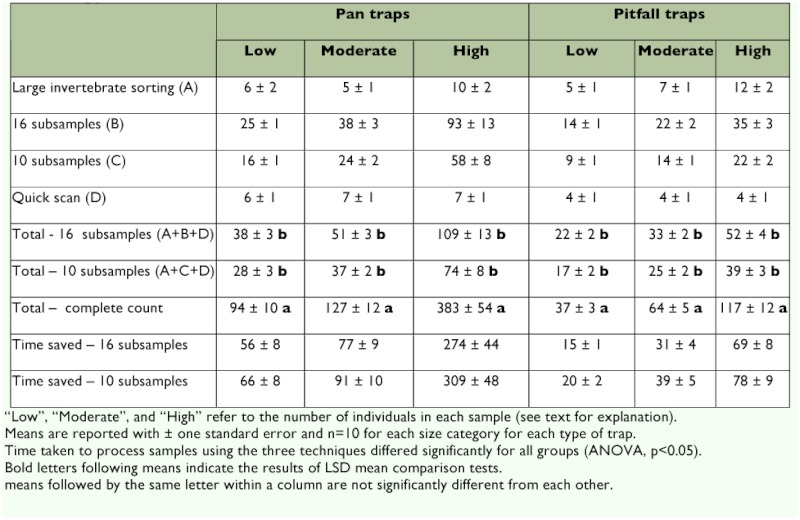
Time (in min) required to process invertebrate samples collected by pan and pitfall traps through subsampling and total counting procedures.

For pitfall traps, the first phase for low and moderate abundance samples required 9–22 min and high abundance samples required 22–35 min for sampling 10–16 cells (Table 2). The entire three-phase subsampling procedure took 22–52 min for 16 cell counts and 17–39 min for 10 cell counts (Table 2). This is compared to 37–117 min for counting the entire sample. As with pan traps, the time required to count the entire sample was significantly greater than the time required to process samples using 10 or 16 subsamples for all size categories (Table 2). Taking 16 subsamples saved, on average, approximately 15–30 min per sample for low and moderate abundance samples and over an hour per sample for high abundance pitfall traps compared to complete counting of the entire sample. An additional 5–13 min were saved per sample by taking 10 subsamples instead of 16 (Table 2).

## Discussion

A formidable challenge faced by investigators of terrestrial invertebrate ecology and biodiversity is processing dozens to hundreds of samples, each with potentially hundreds to thousands of individuals. The time involved in processing these samples makes many largescale studies of terrestrial invertebrate communities cost-prohibitive. Aquatic invertebrate ecologists face the same challenge and have developed subsampling techniques designed to reduce the time required to process large samples of invertebrates while maintaining accuracy in estimates of abundance and taxa richness (Vinson and Hawkins 1996; Walsh 1997; Somers et al. 1998; Doberstein et al. 2000). In contrast, little information is available relative to the effectiveness of subsampling techniques for terrestrial invertebrate samples including descriptions of an effective subsampling apparatus and laboratory technique and data on the precision, accuracy, and time-savings associated with such a technique. We are aware of only one study that examined a form of laboratory subsampling for terrestrial invertebrates; Corbet (1966) described a technique used to estimate abundance of large samples of Trichoptera adults collected with light traps. His technique was aimed primarily at estimating changes in abundance in common Trichoptera species. He made no comparisons of how well his technique estimated the true abundance or taxa richness of the larger sample, and he presented no data on time savings of subsampling.

The results of this study illustrate how a threephase subsampling technique that involves (1) retaining and sorting large specimens, (2) taking random subsamples using a specially designed subsampling apparatus, and (3) quick scanning of the remainder of the sample can be effectively used to address research questions primarily concerned with terrestrial invertebrate abundance (number of individuals) and/or questions of broad taxa richness. Importantly, this subsampling method resulted in significant time savings with little compromise in the accuracy of abundance and taxa richness estimates for moderate and high abundance samples. In this study, 60 samples, which varied in abundance from 93-5,337 individuals each, were examined from pan and pitfall traps. Complete counting of high abundance samples from pan traps took approximately 6.4 h, and the largest samples required more than 12 h to process the entire sample. On average, more than 4.5 h of processing time per sample was saved when the subsampling method was used on these samples. Also, significant time savings of 1–1.5 h were associated with subsampling low and moderate abundance pan trap samples. Subsampling pitfall traps also resulted in time savings, although the amount of time saved for low and moderate pitfall samples was less than it was for pan traps. Nevertheless, subsampling high abundance pitfall trap samples resulted in a substantial decrease (> 1 hour per sample) in processing times.

It is important to note that these are time savings associated with the processing of individual samples, and thus must be interpreted in the context of the average number of samples associated with a typical study. Frequently, studies examining questions of ecological and conservation interest can easily involve hundreds of pitfall and/or pan traps, making the potential for indepth studies virtually impossible. For example, in this study, pan trap samples were taken from a two-year study that involved 14 riparian areas sampled eight times each year. Each site had four pan traps, resulting in a total of 896 pan trap samples. If one-third of these samples were low abundance, one-third were moderate abundance, and one-third were high abundance and the entire samples were processed, it would take 1.4 work years to process these samples to order or common families. This estimate does not include other time-consuming components of processing such as initial sample preparation, recording, labeling, and further identification. Using the subsampling technique suggested here for moderate and high abundance samples (and using whole counts for low abundance samples) would take only 0.43 work years (or 31%) as long) for the example given above. These time savings will change proportionally to the ratio of moderate and high abundance samples.

An important factor to weigh against time savings associated with a subsampling procedure is its accuracy. This study showed that the subsampling technique estimated abundance relatively accurately and precisely for moderate and high abundance samples. However, the abundance of invertebrates in low abundance pitfall trap samples was overestimated by approximately 20%. This margin of error is fairly large and the time savings were relatively small for low abundance samples; therefore, subsampling low abundance samples is not recommended.

The subsampling technique also appeared to provide a good estimate of broad scale taxa richness. All three-phases of the sorting process -- retaining and sorting large specimens, taking random subsamples, and quick scanning of the remainder of the sample -- provide information for taxa richness estimates. The first phase is important in detecting large, sometimes rare, taxa, and it also aids in the uniform distribution of the remaining invertebrates inside of the subsampling tray. Many aquatic macroinvertebrate protocols have a similar step (often called a “large-rare search”) for the purpose of improving estimates of taxa richness (e.g., Gerritsen et al. 2000; Carter and Resh 2001; King and Richardson 2002). The third phase, quick scanning, aids in identifying small, relatively rare taxa that are an important component of taxa richness. This step is not used in aquatic macroinvertebrate subsampling techniques because the amount of substrate associated with the typical benthic macroinvertebrate sample is large, making a visual scan of this type unproductive. In contrast, samples from pan and pitfall traps have relatively little substrate, and taxa not found in the second phase can be detected in the third phase and used to improve taxa richness estimates. The combination of these three phases resulted in, on average, less than two taxa being missed using the 16 cell subsampling procedure compared to the whole counting process. Except for low abundance pan trap samples, in which only 82% of the taxa were detected, the three-phase sampling technique detected 87–93%) of the taxa present in a sample.

Another objective of this study was to determine how many subsamples maximize the information obtained from each sample while minimizing the time involved in processing. High accuracy for moderate and high abundance samples was achieved after even as few as six subsamples. In general, the accuracy of abundance estimation did not change substantially after 8–10 subsamples were taken for both pan and pitfall trap samples. On average, taxa richness increased rapidly during the first 8 subsamples but the rate of increase slowed when taking 9–16 subsamples. Reducing the number of cells subsampled may result in a less accurate estimate of taxa richness; on average, two additional taxa were detected when taking 9–16 subsamples. However, it is likely that the missing taxa would be detected during the quick scanning procedure. Nevertheless, in studies where detecting small differences in taxa richness are important, taking up to 16 subsamples is recommended. The need for accuracy must be weighed against the potential time-savings. In this study, taking 10 subsamples instead of 16 saved between 8–36 min for pan trap samples and 3–9 min for pitfall trap samples (Table 2).

This research also aimed to determine whether the technique was associated with any biases in taxa detection, such that certain taxa were more prone to be missed in the subsampling process than others. In general, taxa that were relatively rare tended to be missed more often than common taxa. For example, although Trichoptera larvae were not detected with the subsampling technique in any of the pan trap samples, they were only present in three of the 30 samples. However, a few taxa were fairly common and were frequently missed in pan trap samples, including Cercopidae, Aphidae, and spiderlings, which were not detected in 29–36% of the samples (Table 1). Two factors probably contributed to the tendency to miss these taxa — size and body color. Individuals of these taxa are not only small, but are also light colored, and thus were difficult to see against the white background of the sampling tray. Thus, when using subsampling techniques, particular care should be taken when dealing with small specimens that blend into the background. If these taxa are common or of particular interest, more effort can be taken to develop a search image for these taxa, or a different colored sorting tray (e.g., black) can be used so that the taxa are more noticeable.

Another question of interest is whether the effectiveness of the subsampling method varied depending on whether the sample was collected with pan traps or pitfall traps. There were several important differences between the two types of samples. Pitfall trap samples generally had fewer invertebrates than pan trap samples, especially for high abundance samples. There were also differences in the size and condition of invertebrates in the two types of samples. Pitfall traps contained, on average, larger and better preserved invertebrates than pan traps. In addition, because the pan traps were fairly large and received a relatively high amount of sunlight, many samples had algal growth, which tended to entangle invertebrate specimens. All of these factors resulted in longer processing times for pan traps compared to pitfall traps, and thus, the time savings associated with subsampling pitfall samples was reduced compared to pan traps. In general, then, longer processing times may be needed when a collecting method results in smaller, more fragile, and/or algal entangled invertebrates, and time savings associated with subsampling in those cases can be substantial. Another difference between the two types of samples was that more taxa were missed during subsampling of pitfall trap samples as compared to pan trap samples. This pattern may be, in large part, due to the fact that taxa in pitfall samples were identified to a higher taxonomic resolution than taxa in pan trap samples. Pitfall traps also contained larger amounts of substrate (e.g., sand, silt, and other debris) which might obscure small taxa.

There are limitations to the use of this technique. The method was only applied to common forms of terrestrial sampling that result in collections with specimens preserved in liquids. Liquid facilitated the even distribution of invertebrates inside of the sampling plate. Even distribution of samples inside of the sampling tray was very important for accurate abundance estimation. Further tests are needed to examine how the method might be modified to accommodate samples that are not preserved in liquid. The size of the tray could also be adjusted, depending on the typical sample size. For example, a larger sampling tray and divided metal frame could be used to hold extremely large samples.

## Recommendations

In general, the cost and impracticality of processing samples that contain several thousand invertebrates leads to the need of using some type of subsampling procedure to provide an unbiased representation of a larger sample (Barbour and Gerritsen 1996). Using a subsampling apparatus, as described here, is recommended to divide the entire sample into equal subsamples. Subsamples should be randomly selected. After large invertebrates and plant material are removed, the sample should be evenly distributed inside of the sampling tray by agitating and detaching entangled invertebrates using a small brush. This step is particularly important in order to assure uniform distribution of invertebrates in the subsampling tray. Counting the invertebrates in only 10 cells (i.e., ∼16% of the entire sample) provided accurate estimates of abundance and taxa richness; counting additional cells did not appear to increase precision or accuracy of abundance estimates. Whether this level of subsampling provides accurate estimates of abundance and taxa richness for terrestrial invertebrate samples collected using other techniques or collected in other locations still needs to be tested. Because abundance estimates of low abundance samples were not very accurate, subsampling samples that contain <250 invertebrates is not recommended. Thus, if the average number of invertebrates per cell is < 4 after sampling 10 cells, counting the entire sample is recommended. For samples with average densities > 4 invertebrates per cell, the total abundance of invertebrates can be estimated by multiplying by the average number of invertebrate per cell by the number of cells per sampling tray (for this apparatus it is 63.6 cells/per plate). The number of large invertebrates separated from the sample during the first phase is added to this estimate to obtain an abundance estimate for the entire sample. Taxa richness is simply calculated by adding the number of taxa in all three phases (large invertebrate separation, subsampling, and the quick scan).
